# Impact of Underwater Image Enhancement on Feature Matching

**DOI:** 10.3390/s25226966

**Published:** 2025-11-14

**Authors:** Jason M. Summers, Mark W. Jones, Catherine Seale

**Affiliations:** 1 Department of Computer Science, Swansea University, Swansea SA1 8EN, UK; 2 Beam, Bristol BS1 6BX, UK; contact@catherineseale.com

**Keywords:** underwater image enhancement, SLAM, feature matching, furthest matching frame, feature robustness, context-aware benchmarking

## Abstract

We introduce local matching stability and furthest matchable frame as quantitative measures for evaluating the success of underwater image enhancement. This enhancement process addresses visual degradation caused by light absorption, scattering, marine growth, and debris. Enhanced imagery plays a critical role in downstream tasks such as path detection and autonomous navigation for underwater vehicles, relying on robust feature extraction and frame matching. To assess the impact of enhancement techniques on frame-matching performance, we propose a novel evaluation framework tailored to underwater environments. Through metric-based analysis, we identify strengths and limitations of existing approaches and pinpoint gaps in their assessment of real-world applicability. By incorporating a practical matching strategy, our framework offers a robust, context-aware benchmark for comparing enhancement methods. Finally, we demonstrate how visual improvements affect the performance of a complete real-world algorithm—Simultaneous Localization and Mapping (SLAM)—reinforcing the framework’s relevance to operational underwater scenarios.

## 1. Introduction

In recent years, there have been large developments of offshore wind farms (OWFs). These, and other offshore structures and assets, are in greater need of inspection and monitoring compared to structures on land because structures in an underwater environment may be subject to biological growth, turbulent currents, corrosion and physical degradation, all of which may contribute to an increase in fatigue. Checks and maintenance for these structures present many challenges due to the depth and length of the inspections, and so, many surveys are conducted with remotely operated underwater vehicles (ROUVs or ROVs) or autonomous underwater vehicles (AUVs). These vehicles record hours of footage using a range of sensors, particularly optical. This footage is analysed to detect structural damage, either through environment mapping or by identifying specific regions of interest within the footage. Due to the length of the footage, it is useful to move towards the introduction of automatic analysis algorithms.

Current image enhancement research typically evaluates performance using known or synthetically generated ground truths, along with metrics such as PSNR, SSIM, and LPIPS. However, little attention has been given to its impact on downstream tasks like feature matching, camera motion estimation, and trajectory reconstruction. In this work, we address this gap by evaluating enhancement methods through their influence on these downstream applications.

### 1.1. Challenges of Underwater Data

Image enhancement is used to address several challenges presented by underwater images:

Challenge 1—Occlusion and Illumination:

Inconsistencies in visibility can arise from the varying depths, capture angles, sedimentation and properties of the water, which can greatly affect a model’s ability to classify elements of an underwater image [1]. Objects such as bubbles, debris and biological life can often block regions of the footage, potentially causing the inspector to miss key parts of a structure. Light degradation can also be an issue; the Lambert–Beer empirical law states that decay in the intensity of light depends on the properties of the medium through which the light travels; so, the water itself can alter the colour and illumination of objects [2,3]. For every 10 metres of depth underwater, the light available is halved [4]. Visibility can also vary with weather, and storms can create turbulent water, creating a more complex medium that amplifies the issues above. Light degradation and absorption are not constant for all wavelengths of light, longer wavelengths like red and orange are more easily absorbed in contrast to shorter wavelengths like blue and green. Light from the surface is therefore altered with depth, where footage often has a blue-green tint.

Challenge 2—Noise and Distractions:

Underwater scenes frequently encounter various sources of noise. Biological debris, known as marine snow, can cause challenges with backlighting, while live fish and bubbles are prominent elements that can affect deep vision models significantly. Additionally, video and image capture may introduce blurring, video noise, and lens distortion. These factors collectively pose barriers to effective image analysis tasks, including feature or object identification, for both humans and vision models. For vision models being used by the inspector, artifacts can negatively affect the contrast of images, or lead to poor embedding spaces or inaccurate similarity scores in the case of deep models. This challenge is particularly pronounced in video inspections, where ROV footage often spans multiple hours.

Challenge 3—Non-stable Video Capture:

Turbulent water causes the ROV to have unpredictable movement and video capture, hindering the temporal value that video could provide. The stability of footage is an important factor in accessing the trajectory of objects in the footage [5], which, in turn, can affect the feature and object detection capabilities of ML models [6,7].

Objectives:

We set out to answer the question of how we measure enhancement with regard to real-world applications using the following objectives:

1. Identify and analyse the current approaches for image enhancement in the underwater domain.
2. Examine the current metrics used to quantify and compare underwater image enhancements.
3. Propose new measures of feature matching consistency over multiple subsequent frames and furthest frame matching to evaluate enhancement.
4. Incorporate these into a new framework for evaluating the real-world applicability of underwater image enhancement.

Next, we review underwater image enhancement (UIE), including how to improve an image, and how to assess the quality of the improvement. We report classical approaches (Section 1.2), followed by a look into deep learning-based methods (Section 1.3) and cover the methods of quality assessment and image evaluation (Section 1.4).

### 1.2. Classical and Physically Based Approaches for UIE

Despite the rise in interest in deep learning in recent years, we found that non-deep learning-based methods still play a significant role in underwater image enhancement.

Popular methods are histogram equalisation (HE), modifying channel priors, and wavelet transforms. These methods share a common approach of spectral augmentation, in that they manipulate regions across the light spectrum to improve contrast. However, while histogram equalization generally provides enhancement across the entire spectrum, wavelet transforms and channel priors focus on specific regions with more discrimination. Histogram manipulation and equalisation was a focus of many publications [8,9,10,11,12,13,14,15]. A basic form of this is global HE, which applies this method to an entire image using a transformation function derived from the image cumulative distribution function (CDF). Histogram equalisation methods are often described in this field as having limited effectiveness in underwater scenes due to the nature of light degradation [13,16]. Consequently, HE techniques have evolved to address domain specific image characteristics, including resulting from the physical properties of water.

The most common improvement in the literature is to perform HE at different scales to limit the influence of noise. Such methods include Contrast Limited Adaptive Histogram Equalization (CLAHE) [17], an important variant designed to enhance contrast in noisy images. Although much of its early use was in medical imagery, it has garnered interest in the underwater imagery domain, being used as a baseline for multiple experiments in the literature [14,18]. It works by performing HE on tiles of an image while limiting the contrast to prevent over-amplification of noise before being interpolated to form the enhanced image.

Bai et al. [8] adopt another multi-scale approach, applying both global Histogram Equalization (GHE) and local Histogram Equalization (LHE) to address different aspects of enhancement. They then adopt a fusion strategy inspired by [19] to combine the luminance, saliency, and exposure weight maps generated from the equalized components into the final enhanced image.

The idea of using fusion to combine various representations is a popular one across both classical and deep methods [8,20,21,22,23,24,25,26,27]. Ancuti et al. [19] present a multi-scale fusion pipeline that breaks the image into Laplacian and Gaussian pyramids, which are blended at each level. Saliency mapping is considered to improve the visibility of objects in the scene. The outcome demonstrates an effective enhancement technique that offers advantages for industrial computer vision. Severely hazy and unevenly illuminated scenes, including industrial scenes, all sourced from a large selection of cameras, were among those considered during their evaluation.

### 1.3. Deep-Learning-Based Methods for UIE

Deep model training continues to be one of the biggest areas of research in this field, growing rapidly. Some relatively older designs continue to remain relevant but with updated features and techniques being incorporated over time.

The challenge of image enhancement, using deep learning, primarily revolves around the use of image-to-image models. These models typically comprise an encoder, responsible for generating a new representation of the input image, and a decoder, tasked with reconstructing or enhancing the image based on this new representation. These are broadly referred to as encoder–decoder models but many variations exist.

Convolutional Neural Networks (CNNs) are widely adopted for feature extraction, using convolving filters or kernels to capture spatial information within image data. Since their inception, CNNs have been prominent in much of the computer vision field, and remain so for underwater image enhancement, being heavily used in many architectures in the literature [24,28,29,30,31]. Attention mechanisms are a more recent addition to the machine learning arsenal, with their efficacy notably demonstrated in 2017 by Vaswani et al. [32]. In the context of images, a spatial attention mechanism can be employed to prioritize and focus on different spatial regions in an image, according to their relevance to the task at hand. Their full potential is still being realised but we can already see examples in the literature that utilise this mechanism for underwater enhancement [33,34,35,36].

Concerning overall architectural frameworks, it is clear from the literature that generative adversarial networks (GANs) had a major impact and influence on the field. Although GANs are adept at generating realistic data, their general functionality does not inherently facilitate image enhancement tasks. CycleGAN [37] is a design that learns mappings between two different domains to facilitate unpaired image-to-image translation tasks and utilises encoder–decoder feature extraction much like an autoencoder. Multiple studies [24,29,33,35,38,39,40,41,42] build on the idea of GANs by using the discriminator to critique the reconstruction capabilities of an autoencoder. Namely, it will discriminate between undistorted images from the source dataset and distorted images that have been enhanced via the autoencoder.

FUnIE-GAN [43] is a successful example of the utilisation of a multitude of methods. They present a convolutional encoder–decoder system that uses an adversarial loss generated by a discriminator for perceptual image enhancement. The design of the encoder–decoder is a U-Net [44], where skip-connections are used between the mirrored encoder–decoder layers. Alongside a new dataset, they also formulate an ensemble of loss functions to access perpetual image quality on numerous levels. Namely, they use $L_{1}$ loss to access global similarity, a VGG-19 backed content loss function [45,46,47], and, finally, an adversarial loss.

### 1.4. Evaluating Performance and SLAM

Objective quantitative metrics are required for performance evaluation, rather than depending on visual inspection of the enhancement results. Peak Signal-to-Noise Ratio (PSNR) and Structural Similarity Index Measure (SSIM) dominate the literature. Other general image quality metrics included Natural Image Quality Evaluator (NIQE) [48], used by papers such as [28,35,49,50,51], Visual Information Fidelity (VIF) [52], used in [28,49], and Perceptual Image Quality Evaluator (PIQE) [53], used in [28,35,50]. There are also metrics focused specifically on underwater image quality, such as Underwater Image Quality Measure (UIQM) [54], which saw use in [41,49,51,55], and Underwater Colour Image Quality Evaluation (UCIQE) [56], which was used in [41,51,55,57]. These metrics deliver a quick and objective analysis for the quality of an image but lack real-world implications, particularly the effect of model-based image enhancement on downstream tasks.

The process of feature matching is crucial to mapping out environments and structures using optical data, an important procedure for industrial surveys, particularly in underwater environments where structural damage is common, and inspections are needed frequently. While some studies, such as those by Ancuti et al. [19] and Yan et al. [57], have tested enhanced data using local feature matching between images, they have been limited to example pairs of images, rather than testing across multiple frames of a video. This ignores the importance of frame-by-frame relationships and does not represent how the enhancement impacts video-based tasks. There is a lack of literature that conducts a comprehensive comparative evaluation in this fashion and this is something we address in this work. Hidalgo and Bräunl [58] examine the behaviours of feature detection, and matching, in frames sourced from varied noisy underwater ROV videos. They compare SIFT [59], SURF [60], ORB [61], BRISK [62], and AKAZE [63] feature detection methods by recording the average features found by each detector, and the average number of inliers using nearest neighbours and homography between two consecutive frames. In the number of features detected and the number of inliers matched, both ORB and BRISK feature detectors performed the best, with SIFT performance following close behind. They additionally apply two image enhancement algorithms, a fusion filter [64], and a backscatter removal filter [65] to each dataset. They found that the enhancements improved the number of detected features across the board but resulted in very low improvements in inliers for all detectors except AKAZE. We evaluate the impact of further state-of-the-art underwater image enhancement on feature detection over longer duration.

SLAM: Feature matching is a core component of Simultaneous Localization And Mapping (SLAM) for mapping 3D points and path of capture source such as a ROV in an environment. Zhang et al. [18] utilised sequential frame matching and SLAM performance as an empirical assessment of a model. They test CLAHE, Median Filtering (MF), and Dark Channel Prior (DCP) using ORB-SLAM 2, an implementation of SLAM that identifies ORB features [61]. They provide both a practical metric for comparing models and guidance for optimising SLAM performance. This approach warrants further exploration and refinement. Hence, in this paper, we address the problem on two levels: first, by measuring the impact of visual enhancement on sequential frame matching ability, and second, by observing its effect on the complete pipeline, in our case, ORB-SLAM 3.

In conclusion, while existing underwater image enhancement methods focus primarily on visual quality metrics, they overlook the downstream impact on real-world tasks such as frame matching and navigation which are part of algorithms such as SLAM. To bridge this gap, we introduce the following innovations:

- Local Matching Stability: a measure of how consistently features are matched across enhanced frames.
- Furthest Matchable Frame: a metric capturing the temporal reach of reliable feature matching post-enhancement.
- Context-Aware Evaluation Framework: tailored to underwater environments and grounded in well-known and practical matching strategies.
- SLAM-Integrated Benchmarking: demonstrating how enhancement affects performance in an industrial context.

## 2. Materials and Methods

To find the impact of visual enhancement on downstream tasks, we apply a range of enhancement methods from the literature, including deep model methods, on underwater videos and measure their impact on feature matching.

### 2.1. Frame Matching

Frame matching involves identifying and correlating features between two images using feature descriptors. Location differences of matched features can indicate movement of the camera or a scene object. By comparing multiple matched features with similar displacements, the camera’s relative movement can be deduced geometrically using a homography model. A higher number of features that consistently align with the model increases confidence in the estimation of the camera’s motion. Matched features whose displacements do not align with the model, either due to the object motion, general noise, or occlusion, are ignored. This is commonly achieved using the Random Sample Consensus (RANSAC) algorithm, which iteratively fits the model to subsets of matches and identifies the largest set of inliers, rejecting outliers as inconsistent matches. The sensitivity of this filter is defined by a threshold for the maximum permissible distance (in pixels) between the actual position of a feature and the predicted position by the model during tuning.

**Figure 1:**
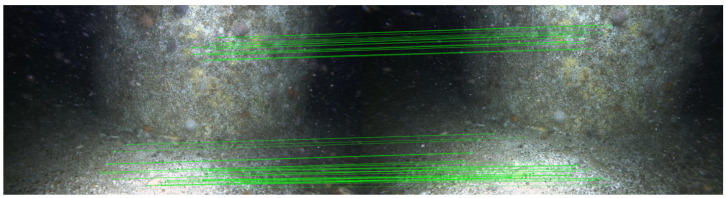
An example of matched features showing ideal feature tracing from two close frames in the ‘Seabed’ video.

In order to test if an enhancement better facilitates frame matching in a video, we employ two functions. One tests how a given frame in the video matches with the next *n* consecutive frames, and the second will find the furthest frame that can still be matched given a set of threshold parameters. The threshold parameters for this study were chosen after experimentation, balancing the number of matched features to avoid both over and under-fitting. The parameters provide a suitable filter for rigorous testing while still enabling successful matches that visibly show aligned, often parallel, feature trace lines during testing, as seen in Figure 1.

#### 2.1.1. Feature Extraction

The main approaches for extracting features are ORB [61], KAZE [66], AKAZE [63], and BRISK [62], being available in the OpenCV library [67]. SIFT [59] and SURF [60] are also important types of feature extraction but have limited availability, particularly SURF, within OpenCV. Superpoint [68] is a keypoint detection system built using a self-supervised CNN framework, and is part of a modern wave of new deep model designs for feature detection and homography [69].

We examine the feature methods SIFT, ORB, BRISK, KAZE, AKAZE, and SuperPoint, omitting SURF due to licensing issues within OpenCV. In order to find sufficient features but maintain an acceptable processing time, we limit the number of extracted features for ORB and SIFT to 1000, the number of octaves to sample for BRISK features to 4, and set the threshold for KAZE and AKAZE to 0.001.

#### 2.1.2. Local Matching Stability

**Figure 2:**
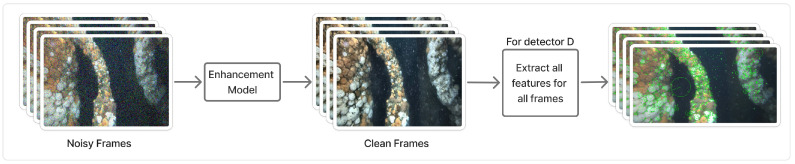
The initial stage of the pipeline, frames are processed with an enhancement model. The chosen feature detector is then used to obtain all features for all frames.

**Figure 3:**
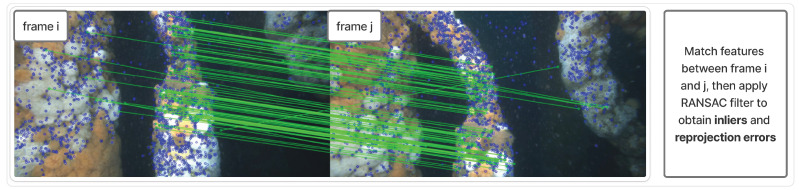
Feature matching process for two frames. The local matching stability and furthest matchable frame measures are computed using information about the matches.

First, we apply a feature-finding technique on every frame in the chosen video (see Figure 2). After extracting all features for all frames, we iterate frame by frame and attempt to match features with the subject frame with those from frame+1, then frame+2, and up to frame+n for the chosen *n*, recording the camera homography data, namely, the average reprojection errors for those features, inlier number, and inlier percentage. We record the matching performance over the n=10 frames from the subject frame (see Figure 3).

#### 2.1.3. Furthest Matchable Frame Metric

In order to compare where enhancement has affected long-term feature matching, we use a similar process to our local matching function, but continue as far as possible given a threshold on the RANSAC filtered the matching results. The parameters used for this threshold are as follows: RANSAC Threshold: 10.0, Inlier Ratio: 0.3, Max Reprojection Error: 20.0. Threshold values were determined through extensive testing on diverse underwater scenes. The selected configuration consistently offered the best balance between feature detection robustness and reliable frame-to-frame matching, maintaining tracking stability even under low visibility and dynamic lighting conditions.

The inlier ratio refers to the minimum proportion of points that must be classified as inliers for the model to be considered valid. We found that this typically low value of 30% was appropriate given the noisiness of underwater data. The max reprojection error measures the distance (in pixels) between the actual and model predicted positions of a feature. However, it is applied to the results of the final model rather than during the RANSAC fitting process. This value is typically closer to or lower than the RANSAC threshold to ensure a stricter more robust model fit. However, due to radial lens distortion, a more lenient reprojection error threshold was used. Frames satisfying these values will yield correctly tracked camera motion with high certainty.

#### 2.1.4. Procedure

The complete procedure for the feature-matching is shown in Algorithm 1. A sequential search is used because we seek continuous matching success; a binary search could be more efficient but would jump past intermediate failures and identify matches that appear later due to loops or repeated scene content, which would misrepresent how long features remain trackable.
**Algorithm 1:** The Complete Feature-Matching-Based Evaluation Framework
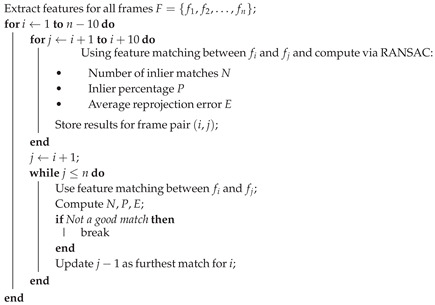


#### 2.1.5. ORB-SLAM 3

As discussed in Section 1.4, visual SLAM is an important procedure in underwater surveys and a key example of frame matching that is used in industry tasks. ORB-SLAM 3 is one implementation that has been frequently used as a benchmark [18,70] and is the state of the art for SLAM implementations using ORB based features. We finalise our testing by observing the performance of SLAM after visual enhancement, using consistent tracking indicators and loop closures as measures.

### 2.2. Test Datasets

**Figure 4:**
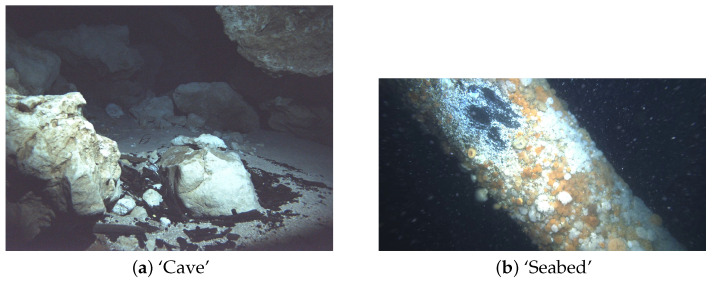
Sample frames from ‘Cave’ [71] and ‘Seabed’ videos.

Our first test video is a publicly available video with loop closures and Inertial Measurement Unit (IMU) data, captured and used in a study by Joshi et al. [71]. This video contains an exploration of a natural cave system. The second test video is sourced from a two-hour inspection of a wind turbine base. This footage was captured using a ROV as it traversed near a variety of pipe structures, including anodes. The footage exhibits many of the highlighted noise forms and additional challenges, such as segments of dark and featureless backgrounds. As a result of these additional challenges, selecting a candidate segment for this test proved to be a difficult task in itself, as segments of quick movement and reduced features prevented consistent completion of the ORB-SLAM 3 process. The desired video should remain challenging, be discriminating amongst techniques and still allow a complete SLAM run to be achievable. We chose a five-minute segment that starts near the seabed and continues up the wind turbine structure, referring to this video as ‘Seabed’. Camera set-up and parameters are in the original work for ‘Cave’ [71]. The ‘Seabed’ video was captured by ROVCO’s SUBSLAM X1 camera featuring a stereo pair of 4 K cameras in a pressure-resistant enclosure (tested to 6700 m). See Figure 4 for representative frames from the two videos.

### 2.3. Test Models

We test models developed by Ancuti et al. [19], FUnIE GAN [43], WaveNet [49,72], WaterNet [31], and, finally, UVENet [73]. This selection includes a variety of approaches, ranging from an important milestone [19] to the state of the art [73], and covers both classical approaches [19,72] and deep models [31,43,49,73].

Each enhancement method is individually applied to each frame of the videos to create the test video set. UVENet [73] highlighted a challenge found among other recent models, particularly those with temporal elements. These models can struggle to process long, high-definition videos. This limitation stems in part from their architectural design and from the limited sizes, both in length and definition, of their training clips. In order to not encounter memory issues while maximising quality, the ‘cave’ video was divided into three parts and individually enhanced. Even so, subsequent upscaling was needed to obtain a definition matching the original.

## 3. Results

### 3.1. Classic Enhancement Validation

An initial analysis was conducted using classic metrics, namely, PSNR and SSIM, to provide a baseline for our results and ensure the enhancement pipeline was functioning correctly.

These values (Table 1), particularly the SSIM scores, are comparable to those from the original research papers, and overall suggest successful enhancements by each of those methods on these challenging new datasets. Based on these values, Waternet [31] performs the best.

### 3.2. Frame Matching Benchmark

Each video is preprocessed by the appropriate feature detection and description algorithm (AKAZE, BRISK etc.). For example, ORB took approximately 25 min to process 6030 frames on an Intel Core i9-12900k with 128 GB, corresponding to a throughput of approximately four frames per second. The entire feature matching evaluation (Algorithm 1), took 210 s at a frame rate of approximately 28 fps.

After running our measures discussed in Section 2.1, we have, for each subject frame, a furthest matchable frame (FMF) value, and the number of inliers, inlier percentage and the average reprojection error for the next ten frames after each subject frame.

#### 3.2.1. Inlier Decay

**Figure 5:**
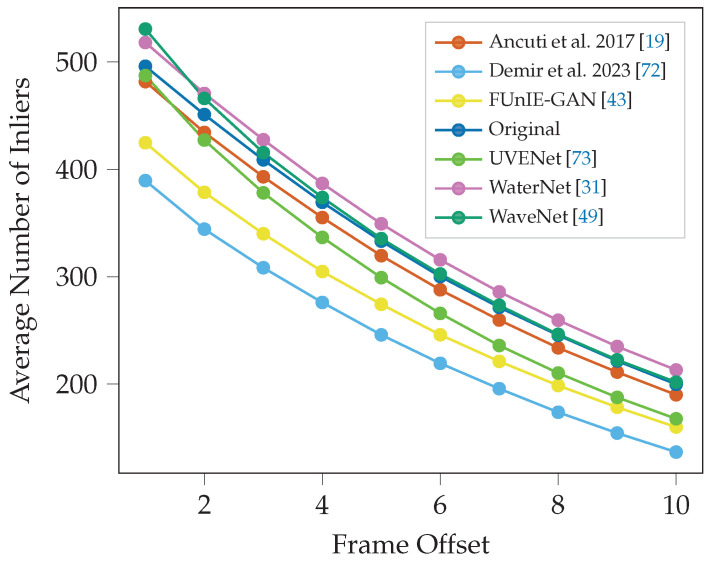
Average number of inliers across subsequent frames offset (1–10) from the subject frame for ORB detector in the Cave dataset.

Figure 5 shows the mean number of inliers for the next ten frames across the whole video for each enhancement method using the ORB detector. The decay curve demonstrates that the next frame (1) has the highest number of inlier matches, and each subsequent frame has fewer matches with the subject frame compared to the previous frame. The video enhanced by WaterNet, WaveNet and the unaltered original video perform consistently the best, with WaveNet and the original video being almost tied in Figure 5. The video enhanced by Demir and Kaplan [72] using a sharpening-smoothing image filter with a CLAHE performs the poorest in facilitating inlier detection.

**Figure 6:**
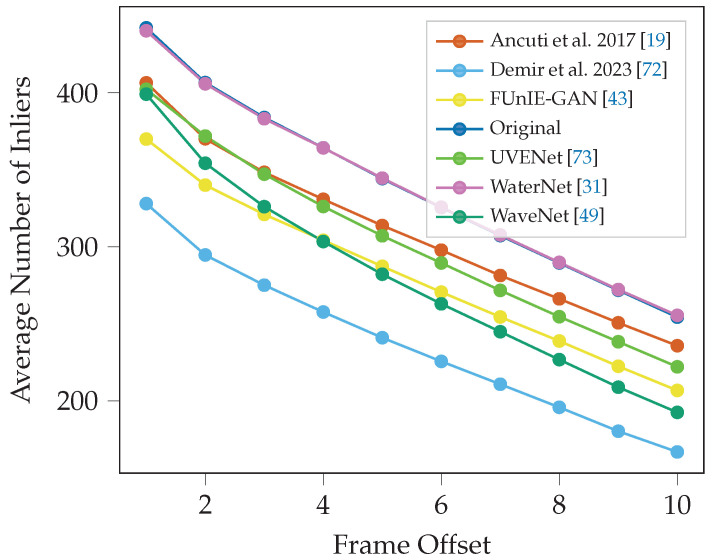
Average number of inliers across subsequent frames for ORB detector in the Seabed dataset. Note that the line for WaterNet (top performer) almost perfectly overlaps the line for the original unenhanced video.

Similar results are seen in the Seabed dataset (Figure 6), where WaterNet and the original video have almost identical results and perform the best. Demir and Kaplan [72] is also the lowest performer. The results of the two datasets are consistent.

#### 3.2.2. Furthest Matchable Frame

The FMF serves as a metric to evaluate how effectively a video enables optimal and consistent feature detection, showing that objects and features have the sustained clarity needed for frame matching for as long as possible. We have two approaches to evaluate the FMF values recorded for each frame. The first is the average furthest matched frame value across the whole video and is presented in Table 2 and Table 3.

We can see in both datasets that videos enhanced by classical methods performed poorly, with Demir and Kaplan [72] coming last in every detector except SuperPoint, and Ancuti et al. [19] coming in the bottom three apart from a few notable exceptions. The unaltered original video for both datasets performed consistently well for all detector types, often coming second. WaveNet, UVENet, and FUnIE-GAN showed varied results, often in the middle rankings, with the one exception of SuperPoint features, where UVENet showed a good improvement over the original video. Only WaterNet showed consistently improved frame matching over all feature types, while all others were consistently outperformed by the original video.

**Figure 7:**
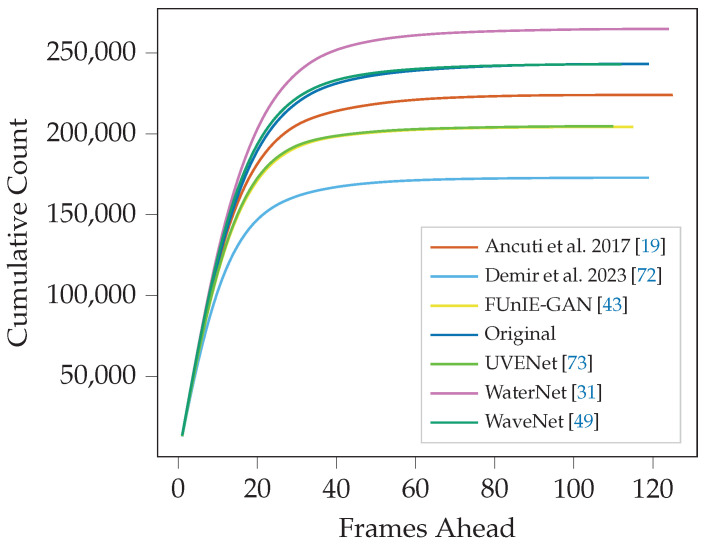
Cumulative distribution of matching distance for ORB feature extraction in the Cave dataset. Higher lines indicate the enhancement, on average, allows frames to match with frames further ahead in the video than lower lines.

**Figure 8:**
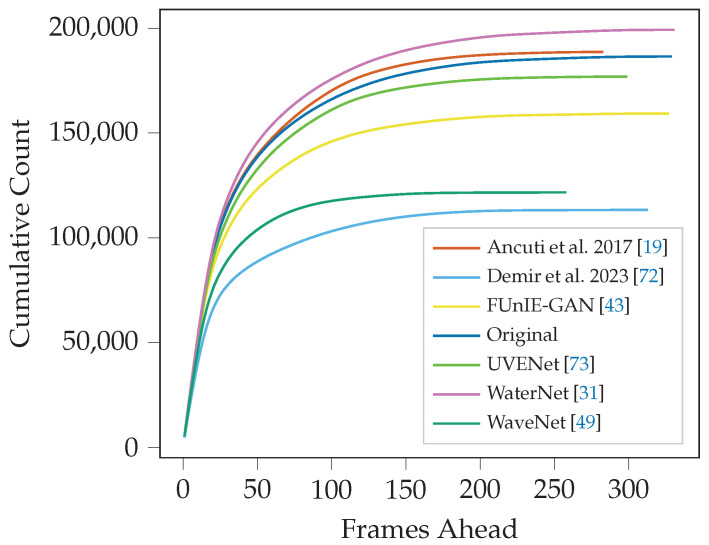
Cumulative distribution of matching distance for ORB feature extraction in the Seabed dataset. Higher lines indicate the enhancement, on average, allows frames to match with frames further ahead in the video than lower lines.

Figure 7 and Figure 8 underscore much of our previous findings. Demir and Kaplan [72] starts to plateau at the lowest values for frames ahead for both datasets, demonstrating an inability to facilitate significantly far matches compared to all other methods. Ancuti et al. [19], UVENet, and WaveNet demonstrate inconsistent results between the datasets. Importantly, WaterNet again performs consistently well on both datasets and is the only method to show a marked improvement in the original video frames.

### 3.3. ORB-SLAM Findings

Finally, we tested the impact of enhanced videos against the original in a full SLAM pipeline. We ran each enhanced or original video from both datasets through ORB-SLAM 3 and recorded tracking status, camera trajectory, loop closure logs, and 3D point cloud data. We found that although ORB-SLAM 3 was able to maintain good tracking for most of the original video, all enhanced videos resulted in significant tracking loss with poor or no recovery once tracking was lost. As a result, each enhanced video showed evidence of trajectory drift and no video, except the original, established any loop closures. Comparing the number of points mapped with the number of frames successfully tracked showed a more even result, with Ancuti et al. [19] doing the best in both datasets, and the original video still doing better than many of the enhanced videos, as well as having the more complete final 3D cloud. These results are presented in Appendix B.

## 4. Discussion and Conclusions

Although the SSIM and PSNR scores indicate that the selected enhancement methods perform without worsening the quality of the original test videos, our findings reveal that the impact of these enhancements are not positive. Testing six distinct approaches, including classical and deep-learning-based methods, we found a wide range of frame-matching abilities. In the majority of cases the videos produced after enhancement were equal or worse in performance compared to the unaltered original video. Demir and Kaplan [72] stood out as performing the worst in our evaluation, while WaterNet [31] was the best and the only method that reliably facilitated better frame-matching results, although only marginally. All enhancement methods tested were detrimental to SLAM’s tracking accuracy, and loop closure detection, leading to drift and incomplete map generation.

Evaluating enhancement performance using full SLAM is computationally prohibitive, particularly for large-scale video data due to significant memory demands. Our central insight is that frame matching—a core component of SLAM—can serve as an effective surrogate metric. This approach offers substantial computational efficiency while still yielding reliable and robust results for cross-method comparison.

Our experiments indicate strong correspondence between frame-matching metrics and the visual trajectories produced by full SLAM, yet the computational overhead is markedly reduced. Consequently, our evaluation framework provides a lightweight, scalable solution for benchmarking underwater image enhancement methods in the context of downstream applications. It offers a practical mechanism to assess whether new enhancement techniques can meaningfully support tasks such as SLAM-based localisation and navigation.

However, there are opportunities to deepen this analysis by incorporating more detailed information on SLAM or other relevant algorithms. Doing so may shed light not only on what causes negative impacts from visual enhancements, but also on why these impacts occur. Key point persistence information provided by ORB-SLAM 3 could be utilised in the future to measure feature clarity consistency on a frame-by-frame basis, forming another metric for enhancement performance. Additionally, there are other enhancement styles yet to be explored, such as temporal models, which fully utilise the information inherent in video data. There are also other industrial algorithms like 3D reconstruction, which have unique challenges not fully addressed in this work.

This framework opens opportunities for future work through the potential use of local matching stability as a component of a loss function in a deep enhancement model. This, managed by a perceptual loss, could ensure that learned improvements promote real and easily matched features across frames, promoting more effective and accurate mapping, although the computation would need to be sped up to enable use for training loss versus validation loss.

## Figures and Tables

**Table 1:** The SSIM and PSNR averages across the Seabed video. Higher is better for both SSIM and PSNR. Methods indicated by † are physically based.

Method	SSIM (↑)	PSNR (↑)
FUnIE-GAN [43]	0.8753	21.68
Ancuti et al. [19] †	0.8394	24.15
Demir and Kaplan [72] †	0.8585	22.83
WaterNet [31]	**0.9209**	**24.85**
WaveNet [49]	0.7132	20.72
UVENet [73]	0.6287	17.46

**Table 2:** Average furthest matching frame (FMF) values for the ‘Cave’ video. A value of *k* in the table means that on average for the whole video each frame matches with the *k*-th frame ahead. Methods indicated by † are physically based.

	Detector				
**Enhancement Method**	**AKAZE (↑)**	**BRISK (↑)**	**KAZE (↑)**	**ORB (↑)**	**Super Point (↑)**
Ancuti et al. 2017 [19] †	20.04	15.41	25.03	16.29	20.85
Demir et al. 2023 [72] †	15.29	10.76	20.90	12.57	22.19
FUnIE-GAN [43]	23.98	19.66	27.09	14.85	21.28
Original	24.26	22.93	27.56	17.68	20.31
UVENet 2024 [73]	20.78	22.67	26.59	15.35	**22.23**
WaterNet [31]	**24.53**	**23.58**	**29.30**	**19.25**	20.67
WaveNet [49]	23.86	22.01	27.63	17.67	21.10

**Table 3:** Average FMF values for the ‘Seabed’ video.

	Detector					
**Enhancement Method**	**AKAZE (↑)**	**BRISK (↑)**	**KAZE (↑)**	**ORB (↑)**	**SIFT (↑)**	**Super Point (↑)**
Ancuti et al. 2017 [19]	38.55	51.52	**64.55**	31.28	**36.57**	27.74
Demir et al. 2023 [72]	29.53	41.75	43.84	18.78	20.95	27.51
FUnIE-GAN [43]	38.49	53.08	54.80	26.41	26.55	24.52
Original	41.35	58.47	57.13	30.93	31.49	20.89
UVENet [73]	39.20	55.19	56.09	29.33	24.79	**28.52**
WaterNet [31]	**42.12**	**61.80**	60.21	**33.04**	32.51	21.82
WaveNet [49]	36.49	53.12	49.91	20.17	23.38	22.69

**Figure A1:**
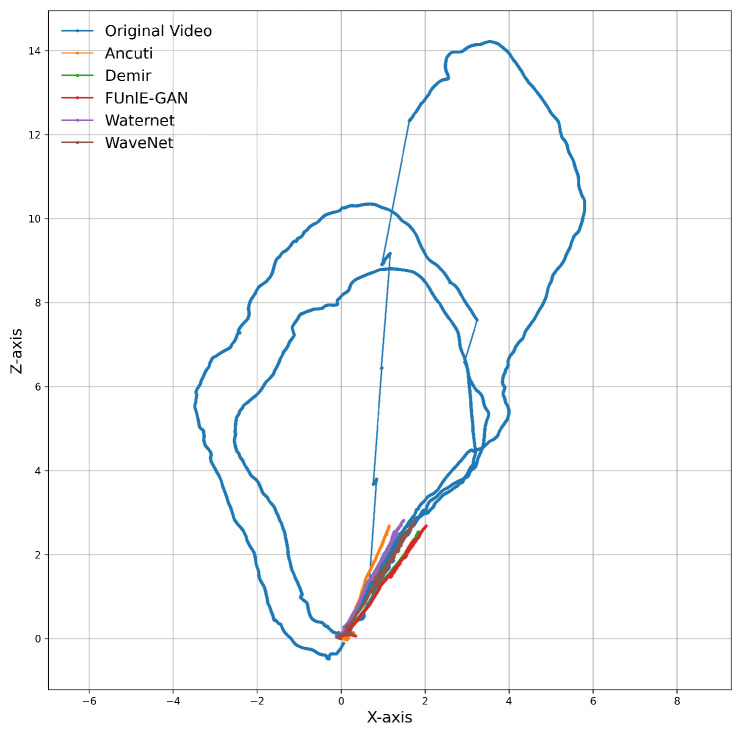
A top–down view of the average path for ‘Cave’ video.

**Figure A2:**
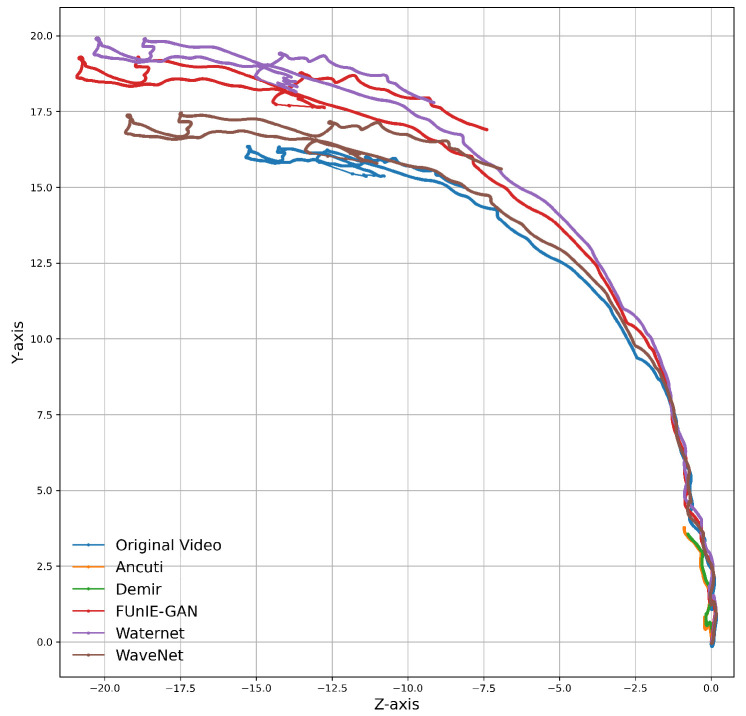
A side view of the average path for ‘Seabed’ video.

**Figure A3:**
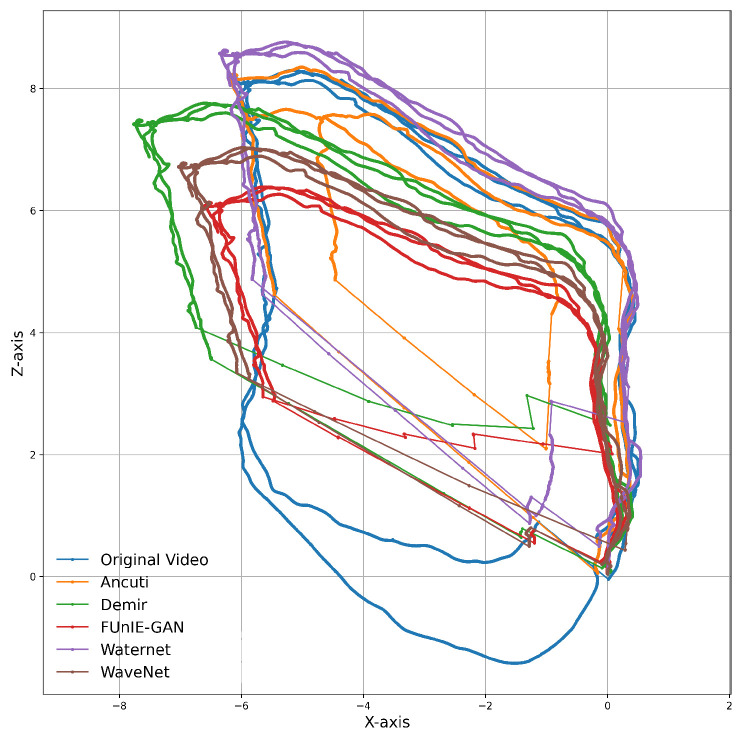
A top–down view of the average path for the ‘Cave’ video started at frame 3500.

## Data Availability

The ‘Cave’ video dataset presented in this study is openly available by Joshi et al. [71]. The ‘Seabed’ video dataset is not readily available because of commercial sensitivity. Code to generate, evaluate and visualise the measures will be made available shortly after publication.

## Appendix A. Additional Data

Table A1 and Table A2 provide specific values for Figure 5 and Figure 6.

**Table A1:** Average number of inliers for different frame offsets across various enhancement methods for the Cave video.

	Average Number of Inliers for Frame Offset:									
**Enhancement Method**	**1**	**2**	**3**	**4**	**5**	**6**	**7**	**8**	**9**	**10**
Ancuti et al. 2017 [19]	481.55	434.28	393.00	355.03	319.56	287.79	259.67	233.78	211.19	190.07
Demir et al. 2023 [72]	389.42	344.26	308.34	276.01	245.87	219.35	195.76	173.81	154.44	136.74
FUnIE-GAN [43]	424.67	378.53	339.95	304.80	274.28	245.92	221.17	198.70	178.44	160.05
Original	495.88	450.94	408.73	369.22	333.09	300.13	271.48	245.50	221.57	199.82
WaterNet [31]	517.97	**470.54**	**427.50**	**386.80**	**349.24**	**315.69**	**285.87**	**259.46**	**235.03**	**213.18**
WaveNet [49]	**530.53**	465.93	415.48	373.71	335.45	302.53	273.40	246.36	222.63	201.85

**Table A2:** Average number of inliers for different frame offsets across various enhancement methods for the Seabed video.

	Average Number of Inliers for Frame Offset:									
**Enhancement Method**	**1**	**2**	**3**	**4**	**5**	**6**	**7**	**8**	**9**	**10**
Ancuti et al. 2017 [19]	406.48	370.15	348.37	330.85	313.80	297.83	281.35	266.19	250.64	235.75
Demir et al. 2023 [72]	328.03	294.69	275.08	257.64	240.95	225.57	210.74	195.76	180.22	166.76
FUnIE-GAN [43]	369.97	340.06	321.03	304.23	287.30	270.58	254.42	238.81	222.39	206.70
Original	**442.21**	**406.62**	**383.94**	**364.30**	344.26	325.53	307.27	289.50	271.79	254.33
WaterNet [31]	440.29	405.84	383.18	364.23	**344.62**	**325.70**	**307.73**	**289.87**	**272.23**	**255.46**
WaveNet [49]	399.12	354.27	326.05	303.41	282.19	262.95	244.83	226.72	208.81	192.47

For example, 495.88 for the original unenhanced video frames in column 1 of Table A1 means that, on average, there will be 495.88 inliers found between frame *n* and frame n+1 throughout the whole (original unenhanced) video. As the offset increases from one to ten, fewer inliers will be found due to camera movement and marine snow and other artifacts in the case of underwater images.

This is a useful and vital measure, because at many-hours-long and 30 frames per second of high-definition footage, SLAM on underwater surveys is highly computationally expensive, and therefore, a way to accelerate the method is to reduce the number of frames. This experiment demonstrates that even three frames per second reduces accuracy and SLAM’s ability to track features.

## Appendix B. ORB-SLAM 3 Results Extended

### Appendix B.1. Motivation

SLAM on underwater imagery is challenging due to the poor visual quality. Therefore, it would seem natural to use enhancement methods on the video frames to improve videos prior to SLAM processing. This was the motivation for this work where SLAM performance is analysed in this section. But it was found that enhancement methods degraded the performance of SLAM over using the original video. There are no available ground truth videos with positional data in an underwater ocean environment (i.e., underwater video with ground truth position, e.g., collected from GPS, in open sea conditions). Therefore, surrogates need to be used to analyse SLAM performance, along with qualitative assessment of paths. We also developed the idea of using feature matching (as the key component of SLAM) as a quantitative measure of enhancement performance, and thus this appears in the main part of this paper, where we can rely on quantitative results rather than the qualitative discussion in this section.

### Appendix B.2. Tracking Ability

We first compared the SLAM tracking ability in the original video with the videos produced from the frames enhanced by each method. We took the average tracking status across five runs of the original and each enhanced video. The tracking status is one of the pieces of data that ORB-SLAM 3 produces per frame, which we log during its operation and belongs to one of three statuses, initialising tracking, OK tracking, and lost/no tracking. The most consistent to register good tracking was the unaltered original video. For both the Cave and Seabed videos, all three deep-learning-based enhancement models managed to regain tracking, while the classical methods, on average, did not regain tracking. We saw variation in the number of frames needed to initialise. For the Cave video, Ancuti et al.’s enhancements proved to be the fastest at initialising, taking an average of 59 frames to start tracking, compared to an average of 370.6 frames for all other cave videos. However, in the case of the seabed video, the classical methods, including Ancuti et al. take the longest by far to initialise.

**Table A3:** The average number of frames to initiate tracking.

	Frames to Initialise	
**Enhancement Method**	**Cave (↓)**	**Seabed (↓)**
Original video	365	**58**
FUnIE-GAN [43]	381	98
Ancuti et al. 2017 [19]	**59**	270
Demir et al. 2023 [72]	380	235
Waternet [31]	357	89
WaveNet [49]	370	74

The poor performance on the Cave video prompted us to test how well each enhanced video would be initialised and tracked within a problematic area indicated by where all enhanced videos lost tracking. We performed this test starting from frame 3500. Despite this area being problematic for SLAM when in the context of the whole video, starting from this region resulted in improved initialisation and better tracking from all enhancement methods. However, the original video remained the only video that resulted in a complete survey, suggesting the enhancements still degraded the performance of SLAM tracking overall.

### Appendix B.3. Trajectory and Loop Closures

The poor tracking performances for the enhanced videos are made more apparent by considering the recorded trajectories from SLAM. Figure A1 and Figure A2 show the average camera trajectory created from each video over five runs.

It is again clear that the original video results in the more complete SLAM survey, showing a clear, sustained path that largely matches the path visually seen in the video. A ground truth is unavailable for comparison as creating a ground truth for underwater imagery is extremely problematic; therefore, expert judgment on the path derived from SLAM, compared to the motion observed in the video is the best qualitative measure that we have. In the case of the enhanced cave videos, we see that no sustained paths were found by SLAM, and therefore there is a substantial difference between the original video and enhanced videos. More interestingly we find that in the case of the Seabed video, where tracking was better, there are sustained paths from all deep-learning enhancements but with clear divergences between them. Given the encouraging tracking performance for the original video, the drift in the enhanced video surveys suggests again that the enhancements are detrimental to SLAM.

Figure A3 shows the recorded camera trajectory when SLAM is initiated in the previously problematic region of the video at frame 3500. The result is a set of more sustained trajectories from all enhancement methods, but again there is strong evidence of drift. The figure shows that all enhancement methods resulted in half a loop before tracking was lost and then regained at the familiar beginning of the loop, preventing a full loop closure. Reinforcing this analysis are the loop closure event logs created by ORB-SLAM 3.

We found that no enhancement method resulted in the capture of a single loop closure in five runs. Only the original video resulted in any loop closure detection, with one event being identified in three of five runs, and in all five when the video started at frame 3500. Despite being unable to complete a loop closure, Figure A3 shows that ORB-SLAM 3 was able to relocalise itself after losing track. This suggests that these enhanced videos may be capable of facilitating loop closures, but as the enhancements lead to inconsistent tracking, the likelihood of loop closure detection is reduced.
